# Supplementary material for: Superspreaders have lower gut microbial alpha-diversity and distinct gut microbial composition in a natural rodent population
Source: Anim Microbiome. 2025 May 6;7:42. doi: 10.1186/s42523-025-00411-1 (PMC12053855; doi:10.1186/s42523-025-00411-1)
Supplement: Supplementary file 1 — Additional file 1. [file 42523_2025_411_MOESM1_ESM.docx]

**Supplementary figures**

**Fig. S1** Elbow plot of cluster distance vs. number of clusters indicates that the optimum number of shedding clusters is four.

**Fig. S2** Elbow plot of cluster distance vs. number of clusters indicates that the optimum number of contacting clusters is four.

**Supplementary tables**

**Table S1** Shedding and contacting clusters and their frequencies in the population of infected voles.

|  | Non-contacter (NC) | Low-contacter (LC) | Intermediate-contacter (IC) | High-contacter (HC) |
| --- | --- | --- | --- | --- |
| Low-shedder (LS) | 73 | 69 | 56 | 26 |
| Low-intermediate-shedder (LIS) | 63 | 61 | 53 | 29 |
| High-intermediate-shedder (HIS) | 47 | 31 | 27 | 13 |
| High-shedder (HS) | 32 | 20 | 30 | 17 |

**Table S2** Shedding and contacting clusters and their frequencies in the population of infected voles with microbiota metadata. Numbers outside the brackets indicate individuals; numbers inside the brackets indicate numbers of faecal samples.

|  | No. individuals (No. faecal samples) |
| --- | --- |
| *Shedding*  Lower-shedder (LS)  Higher-shedder (HS) | 47 (148)  12 (26) |
| *Contacting*  Lower-contacter (LC)  Higher-contacter (HC) | 46 (142)  13 (32) |

**Table S3** Test statistics and *p*-values from likelihood tests of linear mixed effects models testing the association between shedding cluster and various beta-diversity metrics. Bray-Curtis and weighted UniFrac (wUniFrac) distances were calculated and used in Non-Metric Multidimensional Scaling to provide individual scores (wUniFrac: K = 5; Bray-Curtis: K = 3). Robust principal component analysis (RPCA) was also performed and 10 principal components were identified (RPC1–10).

| **Beta-diversity metric** | $\boldsymbol{X}^{\boldsymbol{2}}$ | **Corrected *p*-value** |
| --- | --- | --- |
| Bray-Curtis1 | 0.13 | 0.71 |
| Bray-Curtis2 | 2.07 | 0.32 |
| Bray-Curtis3 | 1.98 | 0.32 |
| wUnifrac1 | 0.00 | 0.98 |
| wUnifrac2 | 2.30 | 0.65 |
| wUnifrac3 | 1.42 | 0.93 |
| wUnifrac4 | 0.61 | 0.98 |
| wUnifrac5 | 0.26 | 0.98 |
| RPC1 | 2.91 | 0.88 |
| RPC2 | 0.00 | 0.95 |
| RPC3 | 0.85 | 0.95 |
| RPC4 | 1.46 | 0.95 |
| RPC5 | 1.11 | 0.95 |
| RPC6 | 2.19 | 0.95 |
| RPC7 | 0.68 | 0.95 |
| RPC8 | 0.19 | 0.95 |
| RPC9 | 0.21 | 0.95 |
| RPC10 | 0.07 | 0.95 |

**Table S4** Test statistics and *p*-values from likelihood tests of linear mixed effects models testing the associations between contacting cluster and various beta-diversity metrics. Bray-Curtis and weighted UniFrac (wUniFrac) distances were calculated and used in Non-Metric Multidimensional Scaling to provide individual scores (wUniFrac K = 5; Bray-Curtis K = 3). Robust principal component analysis (RPCA) was also performed and 10 principal components were identified (RPC1–10).

| **Beta-diversity metric** | $\boldsymbol{X}^{\boldsymbol{2}}$ | **Corrected *p*-value** |
| --- | --- | --- |
| Bray-Curtis1 | 1.80 | 0.52 |
| Bray-Curtis2 | 0.00 | 0.99 |
| Bray-Curtis3 | 1.26 | 0.52 |
| wUnifrac1 | 1.14 | 0.96 |
| wUnifrac2 | 0.00 | 0.96 |
| wUnifrac3 | 0.66 | 0.96 |
| wUnifrac4 | 0.21 | 0.96 |
| wUnifrac5 | 0.76 | 0.96 |
| RPC1 | 0.05 | 0.83 |
| RPC2 | 2.28 | 0.83 |
| RPC3 | 0.16 | 0.83 |
| RPC4 | 0.64 | 0.83 |
| RPC5 | 10.59 | 0.01 |
| RPC6 | 1.54 | 0.83 |
| RPC7 | 1.95 | 0.83 |
| RPC8 | 1.03 | 0.83 |
| RPC9 | 1.24 | 0.83 |
| RPC10 | 2.79 | 0.83 |

**Table S5** OTUs showing strongest representation in principal component 5 of RPCA (RPC5) loading, with the 10 lowest and 10 highest loading values shown.

| **OTU ID** | **Phylum** | **Class** | **Order** | **Family** | **Genus** | **Species** | **RPC5** | **Type** |
| --- | --- | --- | --- | --- | --- | --- | --- | --- |
| OTU3572 | Bacteroidetes | Bacteroidia | Bacteroidales | Paraprevotellaceae | CF231 | Unknown | -0.63 | Low |
| OTU7893 | Spirochaetes | Spirochaetes | Spirochaetales | Spirochaetaceae | Treponema | Unknown | -0.28 |  |
| OTU5405 | Firmicutes | Clostridia | Clostridiales | Unknown | Unknown | Unknown | -0.20 |  |
| OTU13000 | Firmicutes | Bacilli | Lactobacillales | Lactobacillaceae | Lactobacillus | Unknown | -0.15 |  |
| OTU15789 | Bacteroidetes | Bacteroidia | Bacteroidales | ***Muribaculaceae*** | Unknown | Unknown | -0.08 |  |
| OTU7552 | Bacteroidetes | Bacteroidia | Bacteroidales | ***Muribaculaceae*** | Unknown | Unknown | -0.08 |  |
| OTU1749 | Bacteroidetes | Bacteroidia | Bacteroidales | Paraprevotellaceae | CF231 | Unknown | -0.08 |  |
| OTU8889 | Bacteroidetes | Bacteroidia | Bacteroidales | ***Muribaculaceae*** | Unknown | Unknown | -0.06 |  |
| OTU12949 | Bacteroidetes | Bacteroidia | Bacteroidales | ***Muribaculaceae*** | Unknown | Unknown | -0.06 |  |
| OTU13625 | Firmicutes | Clostridia | Clostridiales | Unknown | Unknown | Unknown | -0.05 |  |
| OTU11677 | Bacteroidetes | Bacteroidia | Bacteroidales | ***Muribaculaceae*** | Unknown | Unknown | 0.07 | High |
| OTU6924 | Bacteroidetes | Bacteroidia | Bacteroidales | ***Muribaculaceae*** | Unknown | Unknown | 0.07 |  |
| OTU16258 | Bacteroidetes | Bacteroidia | Bacteroidales | ***Muribaculaceae*** | Unknown | Unknown | 0.07 |  |
| OTU1966 | Bacteroidetes | Bacteroidia | Bacteroidales | ***Muribaculaceae*** | Unknown | Unknown | 0.08 |  |
| OTU9822 | Bacteroidetes | Bacteroidia | Bacteroidales | Unknown | Unknown | Unknown | 0.08 |  |
| OTU7422 | Bacteroidetes | Bacteroidia | Bacteroidales | ***Muribaculaceae*** | Unknown | Unknown | 0.11 |  |
| OTU9987 | Firmicutes | Clostridia | Clostridiales | Lachnospiraceae | Unknown | Unknown | 0.11 |  |
| OTU8590 | Bacteroidetes | Bacteroidia | Bacteroidales | ***Muribaculaceae*** | Unknown | Unknown | 0.14 |  |
| OTU11449 | Bacteroidetes | Bacteroidia | Bacteroidales | Paraprevotellaceae | Prevotella | Unknown | 0.16 |  |
| OTU13258 | Bacteroidetes | Bacteroidia | Bacteroidales | ***Muribaculaceae*** | Unknown | Unknown | 0.43 |  |

**Table S6** Indicator OTUs for shedding and contacting clusters. Most common family among these OTUs, *Muribaculaceae*, in bold; OTUs associated with both shedding cluster and contacting cluster highlighted in grey (LC = lower-contacter; HC = higher-contacter; LS = lower-shedder; HS = higher-shedder).

| **OTU ID** | **Phylum** | **Class** | **Order** | **Family** | **Genus** | **Species** | **Cluster in which more abundant** | **P value, Sidak-adjusted** |
| --- | --- | --- | --- | --- | --- | --- | --- | --- |
| OTU14603 | Firmicutes | Clostridia | Clostridiales | Ruminococcaceae | Oscillospira | Unknown | HC | 0.01 |
| OTU16663 | Proteobacteria | Gammaproteobacteria | Enterobacteriales | Enterobacteriaceae | Escherichia | coli | HC | 0.04 |
| OTU3243 | Bacteroidetes | Bacteroidia | Bacteroidales | Rikenellaceae | Unknown | Unknown | HC | 0.04 |
| OTU650 | Firmicutes | Clostridia | Clostridiales | Ruminococcaceae | Unknown | Unknown | HC | 0.04 |
| OTU6611 | Bacteroidetes | Bacteroidia | Bacteroidales | ***Muribaculaceae*** | Unknown | Unknown | HC | 0.02 |
| OTU806 | Firmicutes | Clostridia | Clostridiales | Ruminococcaceae | Oscillospira | Unknown | HC | 0.02 |
| OTU11616 | Bacteroidetes | Bacteroidia | Bacteroidales | ***Muribaculaceae*** | Unknown | Unknown | LC | 0.01 |
| OTU5504 | Bacteroidetes | Bacteroidia | Bacteroidales | ***Muribaculaceae*** | Unknown | Unknown | LC | 0.02 |
| OTU6618 | Bacteroidetes | Bacteroidia | Bacteroidales | ***Muribaculaceae*** | Unknown | Unknown | LC | 0.03 |
| OTU7357 | Bacteroidetes | Bacteroidia | Bacteroidales | ***Muribaculaceae*** | Unknown | Unknown | LC | 0.01 |
| OTU941 | Bacteroidetes | Bacteroidia | Bacteroidales | ***Muribaculaceae*** | Unknown | Unknown | LC | 0.03 |
| OTU11999 | Bacteroidetes | Bacteroidia | Bacteroidales | ***Muribaculaceae*** | Unknown | Unknown | HS | 0.02 |
| OTU13778 | Bacteroidetes | Bacteroidia | Bacteroidales | Rikenellaceae | Rikenella | Unknown | HS | 0.01 |
| OTU13806 | Unknown | Unknown | Unknown | Unknown | Unknown | Unknown | HS | 0.04 |
| OTU14433 | Firmicutes | Clostridia | Clostridiales | Unknown | Unknown | Unknown | HS | 0.05 |
| OTU14601 | Unknown | Unknown | Unknown | Unknown | Unknown | Unknown | HS | 0.03 |
| OTU17117 | Unknown | Unknown | Unknown | Unknown | Unknown | Unknown | HS | 0.03 |
| OTU2550 | Bacteroidetes | Bacteroidia | Bacteroidales | ***Muribaculaceae*** | Unknown | Unknown | HS | 0.02 |
| OTU2735 | Bacteroidetes | Bacteroidia | Bacteroidales | Unknown | Unknown | Unknown | HS | 0.01 |
| OTU3286 | Bacteroidetes | Bacteroidia | Bacteroidales | ***Muribaculaceae*** | Unknown | Unknown | HS | 0.05 |
| OTU3669 | Bacteroidetes | Bacteroidia | Bacteroidales | ***Muribaculaceae*** | Unknown | Unknown | HS | 0.00 |
| OTU4980 | Firmicutes | Clostridia | Clostridiales | Unknown | Unknown | Unknown | HS | 0.04 |
| OTU6611 | Bacteroidetes | Bacteroidia | Bacteroidales | ***Muribaculaceae*** | Unknown | Unknown | HS | 0.02 |
| OTU7539 | Firmicutes | Clostridia | Clostridiales | Ruminococcaceae | Oscillospira | Unknown | HS | 0.03 |
| OTU11277 | Firmicutes | Clostridia | Clostridiales | Ruminococcaceae | Oscillospira | Unknown | LS | 0.02 |
| OTU16106 | Firmicutes | Clostridia | Clostridiales | Lachnospiraceae | Unknown | Unknown | LS | 0.05 |
| OTU2241 | Bacteroidetes | Bacteroidia | Bacteroidales | ***Muribaculaceae*** | Unknown | Unknown | LS | 0.01 |
| OTU2845 | Firmicutes | Clostridia | Clostridiales | Ruminococcaceae | Unknown | Unknown | LS | 0.04 |
| OTU3551 | Bacteroidetes | Bacteroidia | Bacteroidales | ***Muribaculaceae*** | Unknown | Unknown | LS | 0.04 |
| OTU7147 | Bacteroidetes | Bacteroidia | Bacteroidales | ***Muribaculaceae*** | Unknown | Unknown | LS | 0.02 |
| OTU741 | Firmicutes | Clostridia | Clostridiales | Unknown | Unknown | Unknown | LS | 0.02 |
| OTU9644 | Firmicutes | Clostridia | Clostridiales | Ruminococcaceae | Unknown | Unknown | LS | 0.04 |
